# Nanometric Integrated Temperature and Thermal Sensors in CMOS-SOI Technology

**DOI:** 10.3390/s17081739

**Published:** 2017-07-29

**Authors:** Maria Malits, Yael Nemirovsky

**Affiliations:** Department of Electrical Engineering, Technion—Israel Institute of Technology, Haifa 3200003, Israel; nemirov@ee.technion.ac.il

**Keywords:** CMOS–SOI, lateral diodes, temperature sensors, thermal sensors, semiconductor devices

## Abstract

This paper reviews and compares the thermal and noise characterization of CMOS (complementary metal-oxide-semiconductor) SOI (Silicon on insulator) transistors and lateral diodes used as temperature and thermal sensors. DC analysis of the measured sensors and the experimental results in a broad (300 K up to 550 K) temperature range are presented. It is shown that both sensors require small chip area, have low power consumption, and exhibit linearity and high sensitivity over the entire temperature range. However, the diode’s sensitivity to temperature variations in CMOS-SOI technology is highly dependent on the diode’s perimeter; hence, a careful calibration for each fabrication process is needed. In contrast, the short thermal time constant of the electrons in the transistor’s channel enables measuring the instantaneous heating of the channel and to determine the local true temperature of the transistor. This allows accurate “on-line” temperature sensing while no additional calibration is needed. In addition, the noise measurements indicate that the diode’s small area and perimeter causes a high 1/*f* noise in all measured bias currents. This is a severe drawback for the sensor accuracy when using the sensor as a thermal sensor; hence, CMOS-SOI transistors are a better choice for temperature sensing.

## 1. Introduction

Nanometric partially-depleted CMOS-SOI technology, is now an established technology in a wide range of low-cost, low power and high temperature applications including sensors, high-performance RF, mobile, and mixed-signal chips [1,2,3,4,5,6,7,8]. In spite the enhanced performance of SOI (Silicon on Insulator) devices offered by the buried oxide (BOX) layer, this layer severely impedes heat conduction to the substrate due to the low thermal conductivity of silicon dioxide. Furthermore, the thickness of BOX plays a role in the heat transfer to the substrate since a thinner BOX will allow better cooling of the device from the substrate contact [9]. Therefore, compared to bulk MOS devices, all SOI transistors are more prone to self-heating effects, especially ones with a thick BOX layer.

A rise in chip temperature may degrade the chip performance and decrease its reliability [10,11], hence, thermal design is a critical issue. Continuous thermal monitoring is necessary to reduce thermal damage, increase reliability, and optimize performance. In order to implement effective thermal management, multiple temperature sensors should be placed in strategic chip areas. The desired number of sensors, their exact location, and accuracy depend greatly on system-level requirements, IC packaging, and the cooling system.

An ideal on-chip temperature sensor should be accurate, compatible with the target process, and have reasonable silicon area so that it can be placed non-invasively across the chip without drastically changing the chip design plan. In addition, the power consumption of the sensor should be very low to reduce the power budget overhead of the thermal monitoring system, and to minimize measurement inaccuracies due to self-heating. However, usually there is a distinct tradeoff between the sensor’s accuracy and its area and power consumption, as well as the need for calibration. Calibration requirements significantly affect the cost of the products. Furthermore, a highly accurate sensor consumes more silicon area and power compared to a less accurate one. Hence, it is challenging to generalize the criterions for an optimal temperature sensor, but it is widely known that the design tradeoffs are largely driven by the exact application and system-level considerations.

A number of silicon physical properties are temperature dependent, hence there are a number of CMOS-SOI devices that may be used as integrated temperature sensors. In addition, by adding MEMS or NEMS to CMOS-SOI devices, the temperature sensors may be thermally isolated, thus providing thermal sensors and thermal imaging systems. Since CMOS and its derivative CMOS-SOI are the most mature and prevalent microelectronics technologies and the key to a significant cost reduction, uncooled thermal sensors based on standard CMOS or CMOS-SOI technology have been extensively pursued [5,6,7,8].

This paper reviews the concepts, advantages and limitations of the leading temperature sensors and thermal sensors fabricated using nanometric CMOS-SOI technology. We compare these sensors in terms of sensitivity, linearity, accuracy, calibration needs, area, and the possibility to measure temperature during the chip operation (on-line) in the temperature range of 300–550 K. In addition, the noise characteristics of the sensors are investigated and compared in order to evaluate their performance as the main component of thermal sensors. This study is performed on *standard* CMOS-SOI MOSFETs and lateral diodes commercially available today and fabricated using CMOS-SOI 180 nm and 130 nm processes [12,13]. The specific dimensions, determined by W/L, were designed by the authors.

## 2. Temperature and Thermal Sensors

An integrated temperature sensor produces an output current or voltage that is proportional to the local area absolute temperature. These sensors are usually small, accurate, and have a fast response time.

Unlike temperature sensors, thermal sensors measure the physical quantities by transducing their signals into thermal quantities first and then transducing the thermal quantities into electrical quantities. A thermal sensor operates in three steps:

1. A non-thermal signal is transduced into a heat flow.
2. The heat flow is converted, within the thermal signal domain, into a temperature difference.
3. The temperature difference is transduced into an electric signal with a temperature sensor.

Hence, the basic building block of each thermal sensor is an integrated temperature sensor.

There are various ways to realize a temperature sensor using CMOS technology. The vertical forward biased diode which, in practice, is a part of a parasitic PNP transistor, is probably one of the most commonly used sensors in the semiconductor industry to monitor temperature [14,15] (see [14], p. 294). Its advantage over other types of temperature sensors includes its compatibility with IC technology, low manufacturing cost, accuracy, and reasonable sensitivity over a wide temperature range [14,15,16]. When the diode is forward-biased at a given current and the junction temperature varies, the voltage across the diode shows a linear variation with temperature [16,17].

In [18], a temperature sensor, which consists of three transistors and has quite good linearity for 1.0 and 0.8 µm processes, has been introduced. However, in nanometric technologies and very low-power applications, with 1.8 V or 1 V supply voltage, the sensor’s linearity is degraded. Some research groups have realized temperature sensors using a time-to-digital-converter, or a ring-oscillator, in a 0.35 µm process or below. However, such temperature sensors occupy a large area and consume excessive power at the required sampling rate [19]. In addition, most of these temperature sensors are not compatible with CMOS-SOI technology.

**Figure 1:**
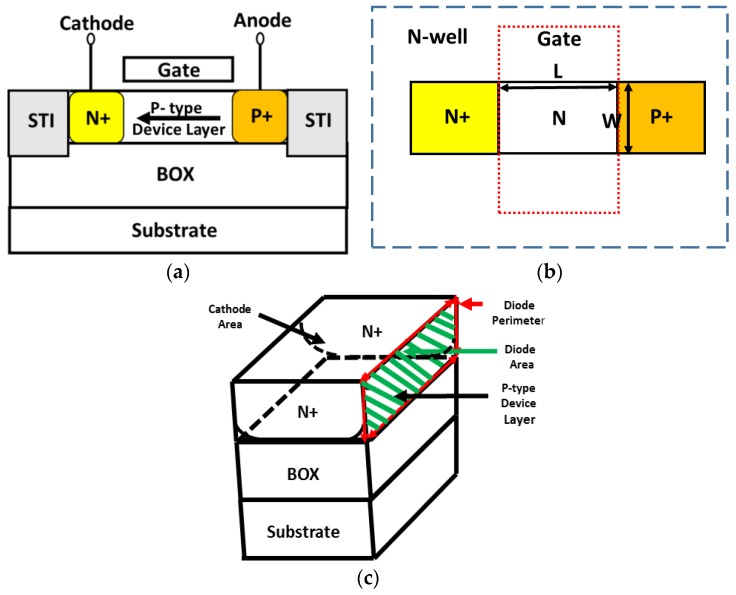
Schematic presentation of a lateral CMOS-SOI diode; (**a**) cross-section; (**b**) overview; and (**c**) cathode cross-section to demonstrate the area and perimeter of the current flow.

The two most commonly used elements for temperature sensing available in CMOS-SOI technology are: standard MOSFET transistors operating at subthreshold levels and lateral diodes [20,21,22,23]. In advanced CMOS-SOI technology it is impossible to manufacture a vertical diode due to the thin body layer; hence, a forward-biased diode is built with a lateral structure based on the device layer, forming a source/drain PN junction under the gate of a MOSFET transistor, as shown in Figure 1a.

Although the SOI lateral diode is modeled as an ideal diode, it should be noted that the saturation current exhibits perimeter dependence rather than area dependence, as in regular planar bulk diodes, due to the thin body layer. Contrary to CMOS bulk diodes, where surface effects may be neglected, the thin device layer in SOI technology requires a model where the current is primarily dependent on surface effects. As a result, the saturation current is strongly affected by the surface, determined by the *device periphery* as shown in Figure 1c. It will be shown that this significantly affects its performance as a temperature sensor and requires a careful calibration process for each technology.

## 3. Thermal Characterization

### 3.1. MOSFET Transistor

In our previous work, we proposed to use the transistor’s threshold voltage (*V_t_*) to determine the local temperature of each chip area [24]. This requires a careful characterization of $V_{t}(T)$ and d*V_t_*/d*T* of the process under study. This part is performed by measuring the current-voltage characteristics of single transistors with relatively small *W*/*L* dimensions in order to avoid self-heating effects, as well as thermal cross-talk between different devices, due to the low power consumption during operation. In addition, the technologies used to fabricate these test devices have a BOX layer, 0.5 µm and 1 µm for the 130 nm and 180 nm processes, respectively, preventing cooling through the substrate and maintaining a constant temperature applied by an external temperature controller during the thermal characterization process.

Subsequently, by monitoring the changes in *V_t_* under actual operation, the true local temperature of larger devices can be determined. We refer to this method as “Threshold-Voltage Thermometry”. It is worthwhile mentioning that this method can be implemented in CMOS technology as well, though the chip temperature rise will be much smaller due to heat conduction to the substrate.

Aiming to evaluate the behavior of MOSFET’s as temperature sensors, commercially-available MOSFETs with different *W*/*L* ratios were designed by the authors and measured in temperatures ranging from 300 K to 550 K. The fabrication was performed in two different SOI partially-depleted technologies [12,13].

The experimental current-voltage curves as a function of the applied temperature have been obtained with an Agilent Technologies (Santa Clara, CA, USA) B1500A semiconductor parameter analyzer and the temperature has been controlled by using a variable temperature micro probe system from MMR Technologies (San Jose, CA, USA), which features a temperature control accuracy of ±0.01 K.

**Figure 2:**
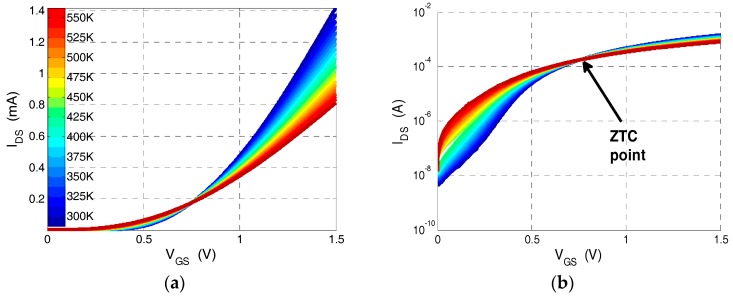
Current-voltage characteristics vs. temperature of an NMOS transistor with *W*/*L* = 43.2/2.4 fabricated using 130 nm CMOS-SOI process: (**a**) on a linear scale; and (**b**) on a semi-log scale.

An example of current-voltage characteristics as a function of temperature for an NMOS transistor fabricated using 130 nm CMOS-SOI process is shown in Figure 2.

**Figure 3:**
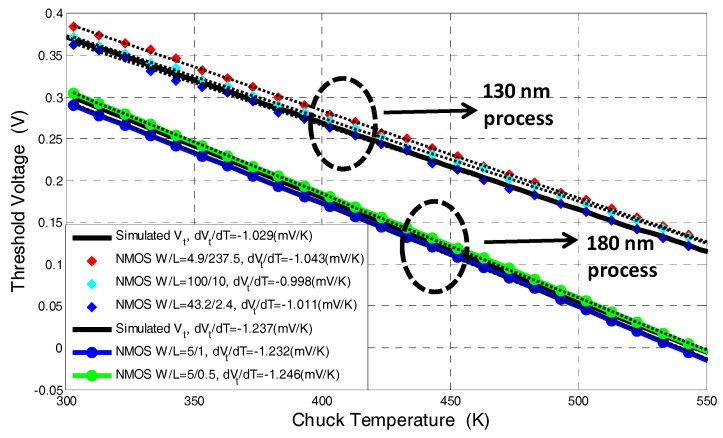
*V_t_* vs. temperature for several NMOS transistors with different *W*/*L* ratios at a temperature range of 300–550 K, which are used to calibrate the processes under study. Dots: experimental data; dashed lines: linear interpolation of the experimental data point; solid: threshold voltage extracted from I-V-T simulation obtained using the SPICE circuit simulator and BSIM4 MOSFET models.

These measurements show the process ZTC (zero temperature coefficient) bias point (*V_GS_* = 0.75 V) which is used to extrapolate the threshold voltage for each temperature. From the results of Figure 2 the *V_t_* vs. *T* curves of several NMOS transistors with different *W*/*L* ratio have been extracted for both technologies and are presented in Figure 3. These results are compared to *V_t_* vs. *T* curves obtained from BSIM4 MOSFET models [25] and electrical simulations in the SPICE simulator.

This calibration curves can be used to convert the extracted *V_t_* into the chip local temperature.

As can be seen in Figure 3, the proposed measurement technique is highly linear over a wide temperature range and its accuracy is determined by the precision of the threshold voltage extraction technique. In order to reduce the temperature dependence of the *V_t_* extraction methodology and increase accuracy, the threshold voltage is extrapolated at the ZTC bias point which is exhibited in Figure 2a,b [24]. The voltage and current at the ZTC bias point are given by [24]:

(1) $$\begin{array}{l} V_{gs,ZTC}=V_{t}(T_{0})-T_{0}(\frac{dV_{t}}{dT})\\ I_{ZTC}=\frac{1}{2}C_{ox}\mu_{o}{T_{o}}^{2}(\frac{W}{L}){\left(\frac{dV_{t}}{dT}\right)}^{2} \end{array}$$

where $V_{gs,ZTC}$ and $I_{ZTC}$ are the voltage and current at the ZTC bias point, respectively, $V_{t}(T_{0})$ is the process nominal threshold voltage, $T_{0}$ is the ambient temperature, $dV_{t}/dT$ is the threshold voltage sensitivity to temperature variations, $C_{ox}$ is the gate oxide capacitance, $\mu_{o}$ is the charge carrier’s mobility in the MOSFET channel, and *W* and *L* are the transistor’s width and channel length, respectively. As can be seen from Equation (1), measuring the ZTC bias points allow to easily obtain the process thermal parameters, i.e., $V_{t}(T_{0})$ and $dV_{t}/dT$, and calculate the process thermal characterization curves.

Figure 3 also presents the threshold voltage sensitivity to temperature variations, i.e., d*V_t_*/d*T* of both technologies. Sensitivities of −1.2 mV/K and −1 mV/K were calculated for the 180 nm and 130 nm processes, respectively. This difference in the two process sensitivities and threshold voltage values are caused by different device layer doping concentrations and different gate oxide thicknesses of the processes under study. These results are in agreement with the simulation results, also presented in Figure 3. It is worthwhile mentioning that this thermal characterization is independent of device dimensions, as can be seen in Figure 3, so it is not easily affected by process variations.

The error in the temperature extraction (Δ*T*) has been obtained by calculating the difference, Δ*V_t_*, between the measured and simulated *V_t_* vs. *T*, which is then converted in temperature using the sensitivity presented in Figure 3. Accordingly, the accuracy is estimated by:

(2) $$\Delta T=\frac{\left|V_{t,measured}-V_{t,simulated}\right|}{dV_{t}/dT}$$

Errors of 3 K and 1 K have been calculated for the 180 nm and 130 nm processes, respectively.

Although careful thermal characterization is needed, i.e., *V_t_* vs. *T* needs to be measured once for each process, the accuracy, linearity and low power consumption required for this method allow it to be used for “on-line” temperature sensing using a “*V_t_* extractor circuit” [26]. Since the significant temperature rise of transistors is induced by thermoelectric effects, and since the thermal time constant of the channel electrons path is very short, heating is almost instantaneous, enabling the on-line measurement of the true temperature of transistors. In addition, due to the small dependence of this method to process variations, no specific sensor calibration process is needed.

### 3.2. Lateral Diode

The current flowing through an SOI lateral diode can be modeled as a forward-biased diode with perimeter dependence [23]:

(3) $$I_{pn}=I_{0}\cdot [exp\left(\frac{qV_{pn}}{n_{f}k_{B}T}\right)-1]\;$$

where *I_pn_* is the diode current, *q* is the electron charge, *k_B_* is Boltzmann’s constant, *V_pn_* is the voltage across the diode, *T* is the absolute temperature, *I*_0_ is the saturation current and *n_f_* is the ideality factor. As emphasized in the introduction, and as shown in Figure 1b, the saturation current *I*_0_ is expressed in terms of the diode perimeter [12]:

(4) $$I_{0}=\;J_{SW}(T)\cdot \;Perimeter$$

where $J_{SW}$ is measured by A/µm and it is temperature dependent.

The temperature dependence of the diode voltage can be derived by rearranging Equation (3):

(5) $$V_{pn}=\frac{n_{f}k_{B}T}{q}[ln\;(\frac{I_{pn}}{I_{0}}\;+1\;)]$$

As seen from Equation (5) there are two temperature-dependent effects changing the diode’s voltage: the increase in the saturation current *I*_0_ and the linear increase of the voltage with temperature. The first effect is much more pronounced; hence, the diode voltage is expected to decrease with temperature when operating at constant current conditions, just like in standard CMOS diodes. The diode sensitivity under forward voltage bias conditions is calculated by differentiating Equation (3) with respect to temperature and assuming *I_pn_* >> *I*_0_:

(6) $$\frac{dV_{pn}}{dT}=\frac{k_{B}n_{f}}{q}[ln(\frac{I_{pn}}{I_{0}})-\frac{T}{I_{0}}\frac{dI_{0}}{dT}]$$

According to Equation (6) the sensor’s expected sensitivity is negative, constant as a function of temperature, and decreases when increasing the bias current.

**Figure 4:**
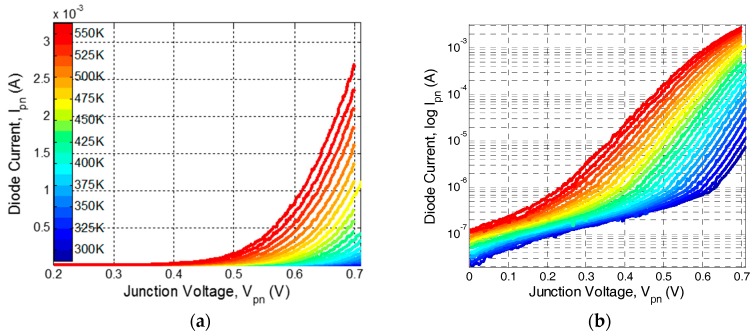
Measured current-voltage characteristics of a lateral CMOS-SOI diode with *W*/*L* ratio of 80 µm/0.6 µm fabricated using the 130 nm process and an area of approximately 48 µm^2^ for different temperatures: (**a**) on a linear scale; and (**b**) on a semi-log scale.

**Figure 5:**
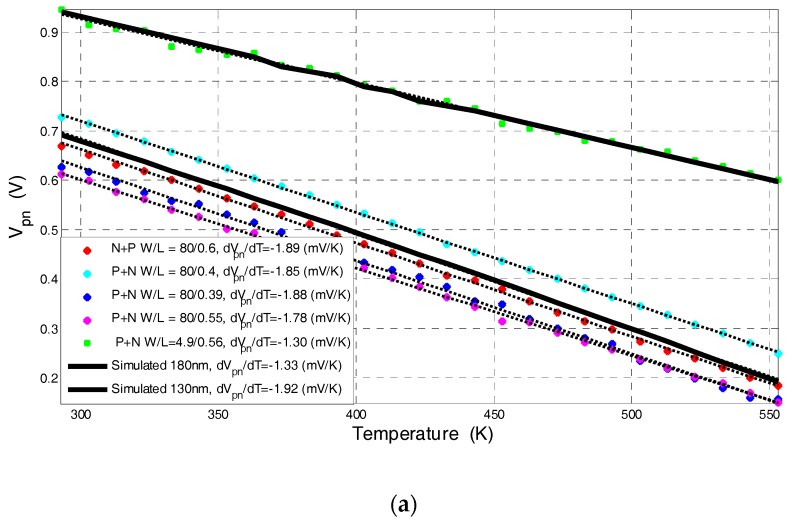

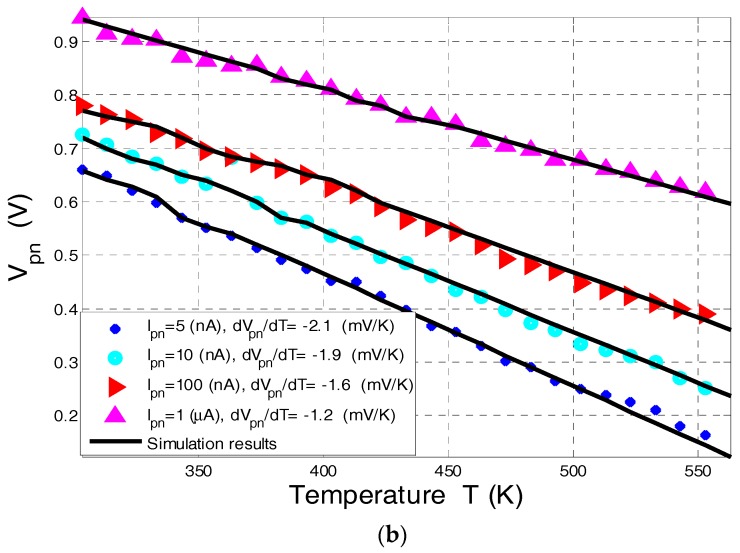
The diode’s forward voltage as a function of temperature (**a**) for different devices at a constant bias current of 1 µA; (**b**) at different bias currents for a lateral diode with *W*/*L* = 4.9 µm/0.56 µm fabricated using the 180 nm CMOS-SOI process. Dots: experimental data; Dashed lines: linear interpolation of the experimental data point; Solid: simulation results.

The current-voltage characteristic of standard and commercially-available lateral diodes with different perimeters fabricated using CMOS-SOI 180 nm [12] and 130 nm [13] processes were measured as a function of temperature in the range of 300 K to 550 K. From these measurements the diode forward voltage was extracted at a constant current point by using Equation (3) for each temperature. Figure 4 presents an example of the measured I-V curves as a function of applied temperature for a lateral diode with *W*/*L* = 80 µm/0.6 µm fabricated using CMOS-SOI 130 nm process [13]. Figure 5 presents the forward voltage extrapolated from the I-V cures shown in Figure 4 as a function of applied temperature. Figure 5a shows different devices fabricated in both technologies at a constant sensing current of 1 µA. Figure 5b shows the forward voltage of a diode fabricated using the 180 nm process at a different sensing current.

As shown in Figure 5, the lateral diode exhibits the same temperature dependence as the standard PN diode implemented in bulk technology in the sense that the voltage decreases as temperature increases. However, since *I*_0_ is dependent on the perimeter of the lateral diodes, the process variations limit the linearity of this method, i.e., the linear behavior of the *V_pn_* vs. *T* curves, to currents above 5 nA, which limits the minimal power consumption needed to preserve the linearity of this method. For example, in the 180 nm process, for the temperature sensitivity of the transistors (ca. −1.2 mV/K), the diode power consumption is at least 95 μW (*I_pn_* = 1 μA and *V_pn, T = 300 K_* = 0.95 V) while, if using a “*V_t_* extractor circuit” [26] to measure the threshold voltage, the circuit power consumption is only ~50 μW. Furthermore, since the diode extracted voltage is highly dependent on device dimensions, a carful calibration process is needed in order to improve the sensor’s accuracy.

**Figure 6:**
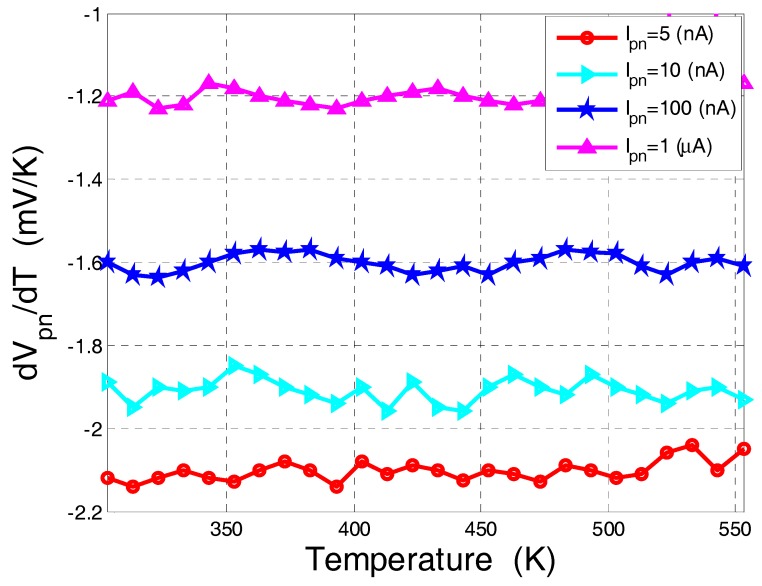
The diode’s sensitivity (d*V_pn_*/d*T*) as a function of temperature extracted from the measured diode presented in Figure 5b.

Figure 6 presents the sensitivity, i.e., |d*V_pn_*/d*T*|, of the lateral diode at different temperatures and different bias currents. These curves show that the sensitivity of the diode increases when decreasing the forward current, as the voltage drop across the neutral regions of the device is less pronounced and the diode’s current is governed by recombination in the space charge region, which is of the order of the leakage current. Citing [23], it should be emphasized that the driving current needs to be low enough to avoid any self-heating while simultaneously providing a high ratio with respect to the reverse saturation (i.e., leakage) current. It can also be observed that the diode sensitivity is negative, constant over the entire temperature range (ca. −2.1 mV/K at *I_DS_* = 5 nA) and higher than the transistor’s sensitivity (~−1.2 mV/K). However, the sensitivity of the diode in CMOS-SOI technology is highly dependent on the diode perimeter and leakage currents; hence, a careful sensor calibration is needed.

The error in the temperature extraction (Δ*T*) has been obtained by calculating the difference, Δ*V_pn_*, between the measured and simulated *V_pn_* vs. *T*, which is then converted into temperature using the sensitivity presented in Figure 6. Accordingly, the accuracy is estimated by:

(7) $$\Delta T=\frac{\left|V_{pn,measured}-V_{pn,simulated}\right|}{dV_{pn}/dT}$$

At a constant current of 1 µA, accuracies of 6 K and 4 K have been calculated for the 180 nm and 130 nm processes, respectively. It is possible to achieve higher accuracy by increasing the sensing current; however, it will increase the power consumption during the measurement.

## 4. Noise Characterization

**Figure 7:**
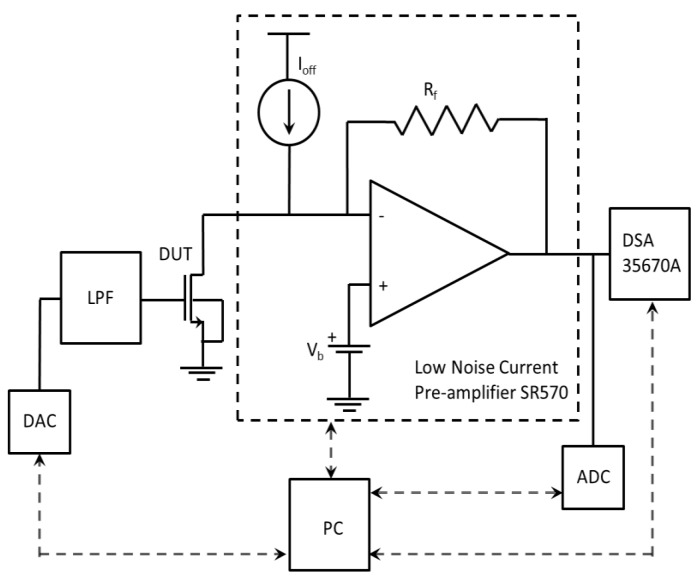
Current noise measurement setup based on a common-source configuration.

The noise characteristics of both sensors were measured in a common source configuration as presented in Figure 7. The measurement setup consists of 35670A dynamic signal analyzer (DSA), low noise current preamplifier (SR570) (including both built-in DC current source (*I_off_*), and a DC voltage source (*V_b_*)), low-pass filter (LPF), ADC, DAC, and a personal computer (PC). The gate of the device is biased by means of DAC, while the drain is supplied by voltage *V_b_* through the current preamplifier virtual ground. The built-in DC current source provides the DUT with the accurate DC current corresponding to the operating voltages so that only current noise is amplified by the current preamplifier and is converted to the voltage noise. DSA measures the voltage noise PSD at the current preamplifier output. The function of ADC is to control the DC voltage at the output of the current preamplifier in order to keep it in a linear regime.

**Figure 8:**
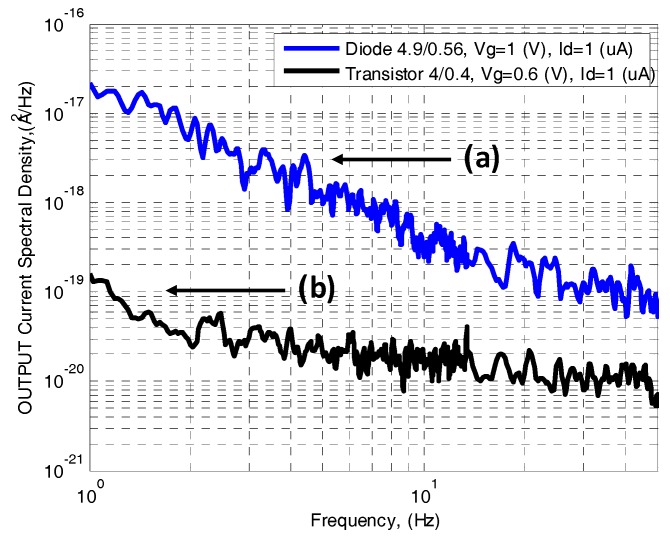
Measured input-referred drain current noise PSD as a function of frequency for a (**a**) lateral diode with area of ~3 µm^2^; and (**b**) NMOS transistor with area of ~2 µm^2^ fabricated with the 180 nm process.

**Figure 9:**
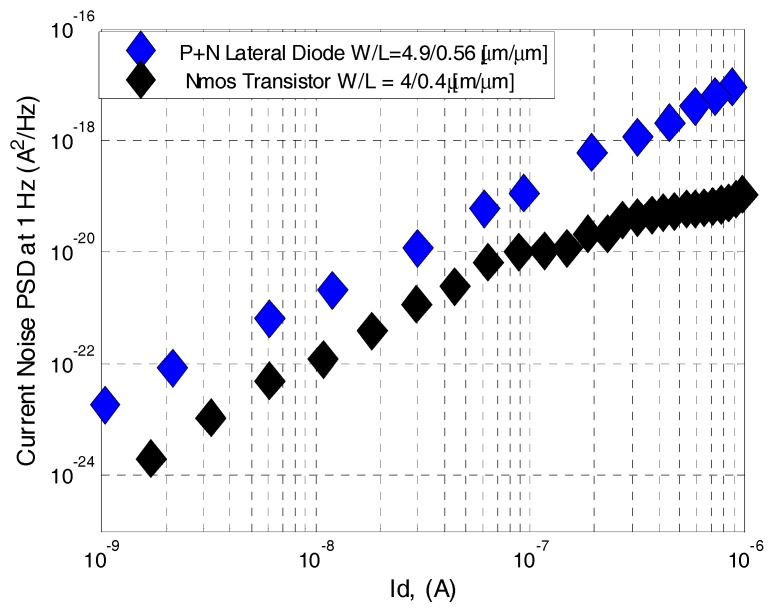
Current noise power spectral density of an NMOS transistor with area of ~2 µm^2^ and a lateral SOI diode with area of ~3 µm^2^.

The noise density spectrum was measured in the range of 1 Hz to 14 kHz for different device currents. The input-referred current noise power spectral density (PSD) is shown in Figure 8 for devices fabricated using the 180 nm process at current level of 1 µA. In both cases the PSD is inversely proportional to the device area; hence, a transistor and a lateral diode of approximately the same area (2 µm^2^ and 3 µm^2^, respectively) were measured and compared in Figure 8 and Figure 9. Figure 9 shows the dependence of the noise current power spectral density on the drain current for the NMOS transistor and lateral diode.

The low-frequency noise of a MOSFET in saturation is calculated using [27]:

(8) $$S_{I,1/f}\left(f\right)=\frac{KF_{sat}\cdot I_{DS}}{C_{OX}L^{2}}\cdot \frac{1}{f}$$

and in subthreshold:

(9) $$S_{I,1/f}\left(f\right)=\frac{KF_{sub}\cdot I_{DS}^{2}}{C_{ox}A}\cdot \frac{1}{f}$$

where *KF_sat_* and *KF_sub_* are the technological noise coefficients in saturation and subthreshold, respectively, *C_ox_* is the oxide capacitance, *L* is the channel effective length, *A* is the transistor area, and *I_DS_* is the transistor DC current.

The PSD of a lateral diode is modeled according to the SPICE model as follows:

(10) $$S_{I,1/f}\left(f\right)=\frac{KF_{diode}\cdot I_{DS}^{\beta }}{A_{diode}}\cdot \frac{1}{f^{\alpha }}$$

where *α* is extracted from Figure 8 and is equal to 1 since we always observed a nearly 1/*f* dependence of $S_{I}$ in the lower frequency range of the spectra. *KF* and *β* represent the SPICE parameters. The study of the device current spectral density evolution at 1 Hz versus device current I_DS_ allows us to extract the value of *β*. Figure 9 shows a quadratic relation of $S_{I}$ at 1 Hz versus *I_DS_*; thus, *β* equals 2.

Figure 9 compares the current noise PSD of the transistor and lateral diode. As seen, between currents of 1 nA to 0.1 µA which correspond to voltages of 0 to 0.25 V at the transistor gate (subthreshold), the lateral diode contributes higher low frequency noise by one order of magnitude. For currents of 0.1 nA to 1 µA which correspond to voltages of 0.25 to 0.5 V at the transistor gate (saturation), the lateral diode contributes higher low-frequency noise by two orders of magnitude.

The noise coefficients (*KF*) of each device were calculated from Figure 9 at different operation regimes. Noise coefficients of $KF_{sat}=6.3\cdot 10^{-29}\;A\cdot F$, $KF_{sub}=1.2\cdot 10^{-21}\;F$ were calculated for the transistor at saturation and subthreshold, respectively, and $KF_{diode}=9.5\cdot 10^{-21}\;{\mu m}^{2}$ was calculated for the lateral diode. The noise measurements indicate that the diode’s small perimeter contributes high 1/*f* noise in all measured bias currents, which is a severe drawback for a thermal sensor.

## 5. Conclusions

In this work the performance of SOI diodes and transistors as temperature and thermal sensors has been presented and compared. Experimental and simulation results of diodes and transistors from two different technologies showed that both sensors exhibit high linearity and sensitivity in a wide temperature range (from 300 K to 550 K). However, SOI transistors provide much more accurate and reliable temperature sensor due to a smaller dependence upon process variations without any additional calibration. In addition, the noise characterization indicates that the current of the lateral diode flows at a small area of diode’s perimeter, resulting in a high 1/*f* noise. The higher noise is a severe drawback when using the sensor as a thermal sensor; hence, CMOS-SOI transistors are a better choice for temperature sensing. In Table 1, the main properties of both sensors are summarized and compared. 

## Figures and Tables

**Table 1:** The temperature/thermal sensors’ main properties.

Technology (nm)	Device	Area (µm^2^)	Maximum Sensitivity (mV/K)	Accuracy (K)	$S_{I}$ (A^2^/Hz)	Calibration
180	Transistor	2	−1.2	3	$1.6\times 10^{-19}$	Not needed
	Diode	3	−2.1	6	$3\times 10^{-17}$	Needed
130	Transistor	50	−1	1	$1.2\times 10^{-21}$	Not needed
	Diode	45	−2	4	$1.4\times 10^{-19}$	Needed

## References

[1] Gianesello F., Giry A., Richard O., Bon O., Boret S., Blanchet F., Boissonnet L., Touret P., Cathelin A., Belot D. Opportunity and Perspectives of using Advanced High Resistivity SOI CMOS technology for the Integration of MultiStandard RF Front-End. Proceedings of the IEEE Power Amplifier Symposium.

[2] François B., Reynaert P. (2015). Highly Linear Fully Integrated Wideband RF PA for LTE-Advanced in 180-nm SOI. IEEE Trans. Microw. Theory Tech..

[3] Ali K.B., Neve C.R., Gharsallah A., Raskin J.-P. (2014). RF Performance of SOI CMOS Technology on Commercial 200-mm Enhanced Signal Integrity High Resistivity SOI Substrate. IEEE Trans. Electron Devices.

[4] Kim J., Dabag H., Asbeck P., Buckwalter J.F. (2012). Q-Band and WBand Power Amplifiers in 45-nm CMOS SOI. IEEE Trans. Microw. Theory Tech..

[5] Svetlitza A., Blank T., Stolyarova S., Brouk I., Shefi S.B., Nemirovsky Y. (2016). CMOS-SOI-MEMS Thermal Antenna and Sensor for Uncooled THz Imaging. IEEE Trans. Electron Devices.

[6] Nemirovsky Y., Svetlitza A., Brouk I., Stolyarova S. (2013). Nanometric CMOS-SOI-NEMS Transistor for Uncooled THz Sensing. IEEE Trans. Electron Devices.

[7] Gitelman L., Stolyarova S., Bar-Lev S., Gutman Z., Ochana Y., Nemirovsky Y. (2009). CMOS-SOI-MEMS transistor for uncooled IR Imaging. IEEE Trans. Electron Devices.

[8] Saraf T., Brouk I., Shefi S.B., Unikovski A., Blank T., Radhakrishnan P.K., Nemirovsky Y. (2016). CMOS-SOI-MEMS Uncooled Infrared Security Sensor with Integrated Readout. IEEE J. Electron Devices Soc..

[9] Pradeep K. (2015). Exploring the Nano-Scale Self-Heating Mechanisms in SOI/Bulk MOS Device.

[10] Monga U., Aghassi J., Siprak D., Sedlmeir J., Hanke C., Kubrak V., Heinrich R., Fjeldly T.A. (2011). Impact of self-heating in SOI FinFETs on analog circuits and inter-die variability. IEEE Electron Device Lett..

[11] Chen J.-H., Helmi S.R., Pajouhi H., Sim Y., Mohammadi S. (2012). A Wideband RF Power Amplifier in 45-nm CMOS SOI Technology With Substrate Transferred to AlN. IEEE Trans. Microw. Theory Tech..

[12] IBM CSOI7RF. http://www.mosis.com/vendors/.

[13] STM H9SOIFEM. http://www.st.com/content/st_com/en/about/innovation-technology/H9SOIFEM.html.

[14] Pertijs M.A.P., Meijer G.C.M., Huijsing J.H. (2004). Precision temperature measurement using CMOS substrate PNP transistors. IEEE Sens. J..

[15] Pertijs M.A.P., Niederkorn A., Ma X., McKillop B., Bakker A., Huijsing J.H. (2005). A CMOS smart temperature sensor with an inaccuracy of ±0.5 °C from −50 °C to 120 °C. IEEE J. Solid-State Circuits.

[16] Mohtashim M., Ibraheem H., Suhail A., Andrea De L., Florin U. (2015). Silicon diode temperature sensors—A review of applications. Sens. Actuators A.

[17] Souri K., Souri K., Makinwa K. A 40 μW CMOS Temperature Sensor with an Inaccuracy of ±0.4 °C (3σ) from −55 °C to 200 °C. Proceedings of the ESSCIRC.

[18] Székely V., Márta C., Kohári Z., Rencz M. (1997). CMOS sensors for on-line thermal monitoring of VLSI circuits. IEEE Trans. Very Large Scale Integr. (VLSI) Syst..

[19] Chen P., Chen C., Tsai C., Lu W. (2005). A time-to-digital-converter based CMOS smart temperature sensor. IEEE J. Solid-State Circuits..

[20] Santra S., Guha P.K., Haque M.S., Ali S.Z., Udrea F. Si diode temperature sensor beyond 300 °C. Proceedings of the CAS 2007 International Semiconductor Conference.

[21] De Luca A., Pathirana V., Ali S.Z., Udrea F. Silicon on insulator thermodiode with extremely wide working temperature range. Proceedings of the 17th International Conference on Solid-State Sensors, Actuators and Microsystems and Eurosensors XXVII.

[22] De Luca A., Pathirana V., Ali S.Z., Dragomirescu D., Udrea F. (2015). Experimental, analytical and numerical investigation of non-linearity of SOI diode temperature sensors at extreme temperatures. Sens. Actuators A.

[23] Sumita S., Prasanta K.G., Syed Zeeshan A., Ibraheem H., Florin U. (2010). Silicon on insulator diode temperature sensor–A detailed analysis for ultra-high temperature operation. IEEE Sens. J..

[24] Malits M., Corcos D., Svetlitza A., Elad D., Nemirovsky Y. (2012). Thermal Performance of CMOS-SOI Transistors from Weak to Strong Inversion. IEEE Instrum. Meas. Mag..

[25] Berkeley Short-channel IGFET Model (BSIM). http://www-device.eecs.berkeley.edu/bsim/.

[26] Fikos G., Siskos S. (2001). Low-Voltage Low-Power Accurate CMOS *V_t_* Extractor. IEEE Trans. Circuit Syst. II.

[27] Nemirovsky Y., Corcos D., Brouk I., Nemirovsky A., Chaudhry S. (2011). 1/*f* Noise in advanced CMOS transistors. IEEE Instrum. Meas. Mag..

